# The Hidden Snake in the Grass: Superior Detection of Snakes in Challenging Attentional Conditions

**DOI:** 10.1371/journal.pone.0114724

**Published:** 2014-12-10

**Authors:** Sandra C. Soares, Björn Lindström, Francisco Esteves, Arne Öhman

**Affiliations:** 1 Department of Education, University of Aveiro, Aveiro, Portugal; 2 IBILI - Institute for Biomedical Imaging and Life Sciences, Faculty of Medicine, University of Coimbra, Coimbra, Portugal; 3 Center for Health Technology and Services Research (CINTESIS), Faculty of Medicine, University of Porto, Porto, Portugal; 4 Division of Psychology, Department of Clinical Neuroscience, Karolinska Institute, Stockholm, Sweden; 5 Department of Psychology, Mid Sweden University, Östersund, Sweden; University of Toyama, Japan

## Abstract

Snakes have provided a serious threat to primates throughout evolution. Furthermore, bites by venomous snakes still cause significant morbidity and mortality in tropical regions of the world. According to the *Snake Detection Theory* (SDT Isbell, 2006; 2009), the vital need to detect camouflaged snakes provided strong evolutionary pressure to develop astute perceptual capacity in animals that were potential targets for snake attacks. We performed a series of behavioral tests that assessed snake detection under conditions that may have been critical for survival. We used spiders as the control stimulus because they are also a common object of phobias and rated negatively by the general population, thus commonly lumped together with snakes as “evolutionary fear-relevant”. Across four experiments (N = 205) we demonstrate an advantage in snake detection, which was particularly obvious under visual conditions known to impede detection of a wide array of common stimuli, for example brief stimulus exposures, stimuli presentation in the visual periphery, and stimuli camouflaged in a cluttered environment. Our results demonstrate a striking independence of snake detection from ecological factors that impede the detection of other stimuli, which suggests that, consistent with the SDT, they reflect a specific biological adaptation. Nonetheless, the empirical tests we report are limited to only one aspect of this rich theory, which integrates findings across a wide array of scientific disciplines.

## Introduction

In his classic treatise on “The expression of the emotions in man and animals”, Darwin [1] recognized snakes as an important evolutionary threat to humans and other primates. He hypothesized that evolution has equipped humans with instinctual responses for handling threatening snakes. In fact, he reported an experiment in which he gave himself the dual roles of experimenter and subject in order to examine whether defense behaviors instigated by a (sham) snake attack were automatically mediated, in the absence of conscious recognition and conscious control. This is his succinct summary of the study:

- *“I put my face close to the thick glass-plate in front of a puff adder in the Zoological Gardens, with the firm determination of not starting back if the snake struck me; but as soon as the blow was struck, my resolution went for nothing, and I jumped a yard or two backwards with astonishing rapidity.”* ([1] p. 43–44).

The work reported in this article took off from Darwin's original observation and from an extensive series of previous work in our laboratories [2]–[7]. The overarching aim of this research program has been to elucidate the interaction between evolutionarily shaped predispositions and specific evolutionary significant stimuli – e.g., snakes, spiders or human faces.

In the present study, we focus on how snakes guide human attention. The rational for the choice of snakes as our critical fear-related stimulus is based on Isbell's [8] Snake Detection Theory (SDT), which is grounded on an erudite argument claiming that snakes over evolutionary time (starting about 100–50 million years ago; see [8], [9], for in-depth reviews) have promoted the development of fear and avoidance of snakes in primates (including humans). We examine the hypothesis that evolutionarily relevant stimuli – snakes in particular – engage different neurobehavioral systems from those activated by more neutral and innocuous stimuli. Specifically, we explore perceptual abilities that may have evolved to facilitate the detection of snakes.

### Beware the snake!

There is no doubt that snakes are perilous creatures that are deadly dangerous to a multitude of living creatures (including primates, and with them humans). Statistics show that the number of human victims whose demise can be attributed to snake bites are of the same orders as – and in fact often exceed – the number victimized by serious tropical diseases [10], [11]. Indeed, “*the yearly mortality caused by snake bites is much greater than that attributed to several presently recognized tropical diseases, including dengue hemorrhagic fever, cholera, leishmaniasis, schistoso miasis, Japanese encephalitis, and Chagas' disease*” ([11] p. 89). Epidemiological estimates of the contemporary yearly death tolls from snakebites suggest that it may approach 100,000 cases worldwide [12]. In addition, about 400,000 humans per year survive attacks by snakes, but many of them may suffer lasting functional impairments [12], [13]. Thus, because snakes have provided such a mortal danger to primates, they likely have served as significant agents of evolutionary selection [9] by eliminating individuals whose defense skills were not effective enough to nullify attacks of snakes or other potentially deadly predators in their habitat.

### Snake detection as a biological adaptation

According to Isbell's Snake Detection Theory (SDT) [8], snakes as predators have had a significant impact on primate evolution. Selection pressures from snakes resulted both in the advanced primate visual system and the common fear of snakes as defensive means for detecting and avoiding snakes [8]. At about 100 mya, early primates were a primary prey for constrictor snakes. At a later stage, the transition between the Mesozoic and Cenozoic (65-30 mya), there was a dynamic period of rapid evolutionary change in many lineages including primates and snakes (“the age of snakes” [14]). During this period snakes developed effective venoms that enhanced the lethal threat they posed to other animals.

Snakes and primates have coexisted in mutual predator-prey relationships for about 100 mya [15], but the extent of exposure has varied across continents, as the result of migration and the breaking up of the southern supercontinent Gondwana [8]. Isbell [8] argues that this variation has produced correlations between evolutionary snake exposure, on the one hand, and fear of snakes and advanced vision in different primate species, on the other. Thus, African monkeys and apes have been continuously exposed to snakes for about 100 my, are uniformly afraid of snakes, and have the most advanced visual system among primates. In contrast, the lemurs of Madagascar, who have a history with very little snake exposure, do not fear snakes and have poor vision. The New World monkeys, finally, who were given a reprieve from snakes that lasted for about 30 my when emigrating from Africa to South America, show variable visual systems and fear of snakes, suggesting less consistent long-term predatory pressure from snakes.

Snakes are carnivorous [14], and use camouflage-based hunting strategies (i.e., ambush predators, [14]–[16]), which involves waiting for prey concealed among rocks, withered leaves or in high grass, an important component in their defense is the breaking of the camouflage by means of superior perceptual skills. Deadly bites by venomous snakes constitute an acute risk also for individuals from non-prey species that happen to come too close to a cryptic snake. Indeed, in an evolutionary perspective, this might be the key to the very advanced visual perception performance of primates, and particularly of the African apes (which includes humans).

Isbell [8] argues that the vital need to detect camouflaged snakes provided strong evolutionary pressure to develop astute perceptual capacity in animals that were potential targets of snake attacks. In mammals, when a threat is detected it is quickly and automatically conveyed to the amygdala, an evolutionary ancient subcortical brain structure, which is the major node in the mammalian fear network, see [17]–[20] for reviews. Thus, snakes may have constituted a primary evolutionary factor promoting the excellent vision – and its functional integration with the fear system – of primates, including the hominines.

### The present study

The work of Isbell [8], [9] outlines the evolutionary contingencies that underlie the effect of snakes on primate vision and attention. In this article we exploit the evolutionary ideas presented by Isbell [8], [9] in seeking to understand the shaping of human perceptual capabilities for efficient detection of snakes as part of the behavioral defense systems.

Previous studies from our laboratory (reviewed in [21], [22]), suggest that snakes are prioritized by the primate attention system. However, most of these results remain inconclusive because separate data for snakes and spiders were typically not reported [3], [23], but were collapsed into a category of “evolutionary fear-relevant animal stimuli”. Furthermore, the findings that snakes and spiders were more effectively detected than control stimuli, flowers and mushrooms, may reflect an attentional bias for animals in general rather than for threatening animals [24].

The emphasis on snakes as a central agent in primate evolution proposed by the SDT [8] provides a novel theoretical rationale for expecting differential effects of snakes and spiders both on human visual attention and defensive behaviors. Spiders are similar to snakes in emotional impact as assessed by ratings of negative valence, high arousal, and dominance [25]. Also, like snakes, they are common objects of phobias [26] and are rated as highly frightening in the general population [27]. Nonetheless, although there are a number of more or less serious symptoms attributable to spider bites, reported mortality is limited [28]. In fact, even for the Australian funnel web spider – considered the most venomous of all spiders – “bites are uncommon and severe envenoming even less common” ([28] p. 485). In addition, because spiders primarily prey on insects rather than mammals [29], the case for an evolutionary origin of spider fear is clearly weaker than that for snakes. Therefore, they are ideal comparison stimuli for testing the SDT.

Currently, few studies have been directly designed to test predictions from the SDT. Van Le et al. [30] showed through single cell recordings in the Macaque pulvinar nucleus that many cells were specifically responsive to snakes, compared to control stimuli. Moreover, Van Strien and colleagues [31] demonstrated earlier capture of visual attention for snakes (compared to spiders and birds), reflected in larger early posterior negativity for this stimulus. However, the experimental paradigm serving as basis for these findings only included passive viewing of the stimuli without any measures of overt or covert visual attention. Two studies from our laboratory have manipulated specific factors depicted from the SDT [32], [33]. The results showed that snakes more potently capture attention than spiders (and mushrooms) under high perceptual load conditions [34]. However, those preliminary studies were designed to open the avenue for the extensive behavioral testing presented in the present study. In the present study, we further increased the perceptual complexity of stimulus displays under several conditions known to deplete attentional resources, to delimitate the conditions where snakes reliably capture attention. Because the SDT holds that snake detection is part of a defense system, with snake avoidance as its evolutionary function, understanding how snakes affect attention on the behavioral level with systematic and robust experimental testing is paramount for examining the SDT [34] predictions.

The objective of the experiments reported in this article was to systematically contrast pictures of snakes and spiders in their effects on human attention. We were particularly interested in examining the modulating effect of ecological factors on the difference between snakes and spiders in capturing attention. The specific factors that we examined were derived from the SDT on the premise that perceptual abilities to detect camouflaged snakes have been more consistently selected for than detection of spiders. Hence, our experimental work focused on factors such as stimulus duration, foveal versus peripheral vision, the complexity of the display as indicated by number of distracting items, and top-down versus bottom-up control of attention, which correspond to ecological conditions thought to be important for snake detection [8].

## Methods

### Participants

Participants were 205 undergraduate students at ISCTE-University Institute of Lisbon (Experiments 1–3) and Karolinska Institutet, Stockholm (Experiment 4), with normal or corrected-to-normal vision: Experiment 1: *n* = 57 (14 males; 17–32 yrs); Experiment 2: *n* = 42 (6 males; 18–32 yrs); Experiment 3: *n* = 57 (12 males; 17–27 yrs); Experiment 4: *n* = 49 (22 males; 18–48 yrs). In order to select a representative sample, the enrolment strategy for Experiments 1-3 opted for a sample of participants with a continuous variation in the fear levels of snakes and spiders, with the aim to reflect the general population. Therefore, we were able to arrange a sample with variable levels of snake and spider fear, i.e., from low to high: Low fear (Exp. 1: 5 males, 13 females; Exp. 2: 4 males, 12 females; Exp. 3: 6 males, 13 females), middle fear (Exp. 1: 6 males, 14 females; Exp. 2: 1 males, 14 females; Exp. 3: 4 males, 16 females), and high fear (Exp. 1: 3 males, 16 females; Exp. 2: 1 males, 10 females; Exp. 3: 2 males, 16 females). This procedure was successful in generating closely similar mean scores on the SNAQ (Experiment 1 = 12.35, Range: 2–28; Experiment 2 = 11.55, Range: 2–28); Experiment 3 = 12.33, Range: 2–28) and the SPQ questionnaires (Experiment 1 = 11.65, Range: 1–27; Experiment 2 = 12.07, Range: 1–27; Experiment 3 = 11.21, Range: 1–27). In Experiment 4 we had a random selection of participants, thus also allowing snake (SNAQ mean score: 4.86, Range: 0–16) and spider fear (SPQ mean score: 4.80, range: 0–16) to continuously vary. Four participants in Experiment 4 were excluded from analyses due to excessively low accuracy (<80% correct in the low load - baseline condition). Participation as subjects in the experiments was based on written informed consent including the right to abort participation at any time. All experiments were approved by the ethics committee at the Karolinska Institutet (2006/77-31) and by the University Institute of Lisbon, who approved the study based on the permit from the Karolinska Institutet.

### Experimental task

In Experiments 1–3, we used different variants of the visual search task [35] to test our hypotheses. Research participants were asked to detect a single member (target) of a stimulus category (snakes, spiders, mushrooms), which differed from a background category containing several pictures (distractors; fruits). The participants were instructed to press different response buttons to indicate if a target (e.g., a snake) was present or absent among the pictures (fruits) shown on a given trial. In this way we could compare detection latencies and accuracy for different types of targets against sets of non-threatening, but ecologically relevant, background pictures [3].

In Experiment 4 a two-choice reaction time task was used and participants were asked to indicate, as quickly and accurately as possible, the identity of a target letter (X or N) while grayscale images of snakes, spiders, flowers and mushrooms were presented simultaneously as distractors on 20% of the trials.

## Experiment 1: Superior detection of snakes with short-duration exposure

Because snakes have a short reactive distance within which they provide serious danger, snake detection should be fast and require only a quick glimpse in order to activate defense. In Experiment 1, therefore, we examined the modulating effect of stimulus duration on detection of snakes in complex scenes. In a previous experiment from our lab [33], the attentional efficiency to detect snakes, compared to spiders and mushrooms, was relatively independent of the perceptual load (set size: 4, 6, 8 items). However, this effect was not influenced by the stimulus exposure durations (150 ms vs. 300 ms). The restricted time for processing targets resulted in lower rates of correct responses and, therefore, in fewer trials on which RTs could be measured, which could have obscured the interaction effects with exposure duration. Thus, it remains unseen if differences between snakes and spiders emerge with a wider manipulation of the exposure duration of the stimulus displays. In the present experiment, participants were exposed to a visual search paradigm in which the duration of the displays varied between 300, 600, and 1200 ms, and the set size varied between 4 and 8 items.

### Apparatus

A 19″ CRT monitor with a resolution of 1024×768 pixels and a refresh rate of 75 Hz, connected to a PC was used for stimulus presentation. The viewing distance was 100 cm. Participants responded by pressing the “yes” (target present – right side) or “no” (target absent – left side) key on a button box.

### Stimuli

The 4 stimuli categories (snakes, spiders, mushrooms, and fruits) each consisted of 18 different color picture exemplars, which displayed the object in center of the picture against a background of its typical ecology. More details about the stimuli are given in S1 File.

**Figure 1:**
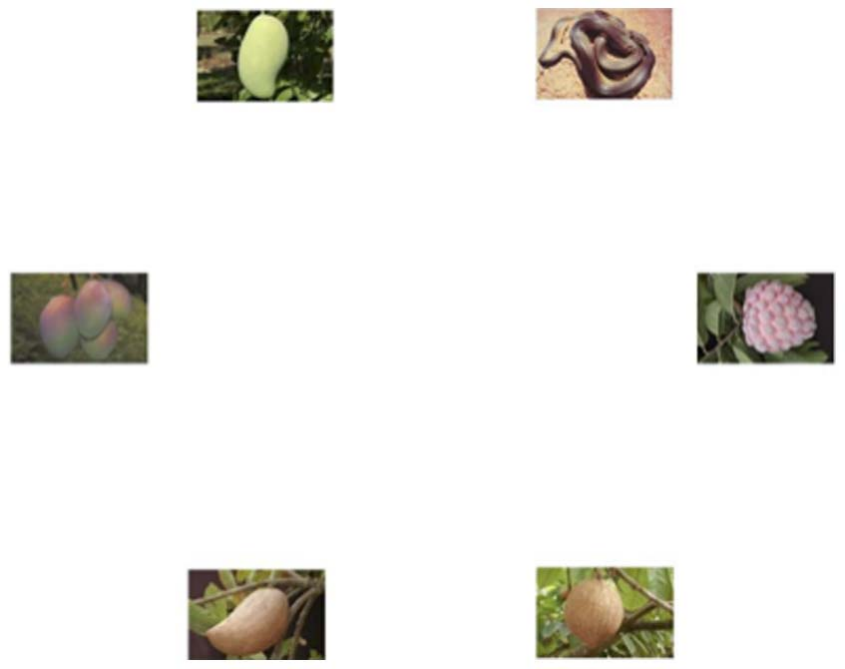
Example of the circular stimuli display (set size 6) used in Exp. 1 and 3.

The pictures in the visual displays were arranged along an imaginary circle around the fixation point with the radius of the circle being the same for the different set size conditions (Fig. 1). The size of the whole display on the screen was 26.0×25.0 cm and each individual picture on the screen extended 3.5×2.3 cm. The distance from the fixation point to the center of each picture was 11.5 cm.

### Task and Procedure

After providing written informed consent, the participants were asked to find a position in the chair where they could comfortably reach the two response keys with their right and left index fingers. Written instructions were self-paced and emphasized that the participant's task was to determine, as quickly and accurately as possible, whether a deviating target stimulus (a snake, a spider, or a mushroom) was present or absent among the background stimuli (fruits). The specific identity of the target stimulus was not specified. A target stimulus was presented on half of the trials in the experiments, whereas the other half only contained distractor displays, where no deviating target stimulus was presented. Target location and order of presentation was randomized for each subject.

Participants first performed a series of practice trials (including displays with and without a target picture). Each trial began with the presentation of a fixation cross (1 cm×1 cm) at the center of the computer screen for 1000 ms, followed by the presentation of the stimulus display, until the participant's response. A 2000 ms inter-trial interval occurred until the reappearance of the fixation cross, initiated the new trial. Participants were exposed to 288 trials (displays with and without a target). Stimulus duration varied between 300 ms, 600 ms, and 1200 ms on different trials. The stimulus set size was either 4 or 8 pictures.

### Results and Discussion

#### Reaction time

**Figure 2:**
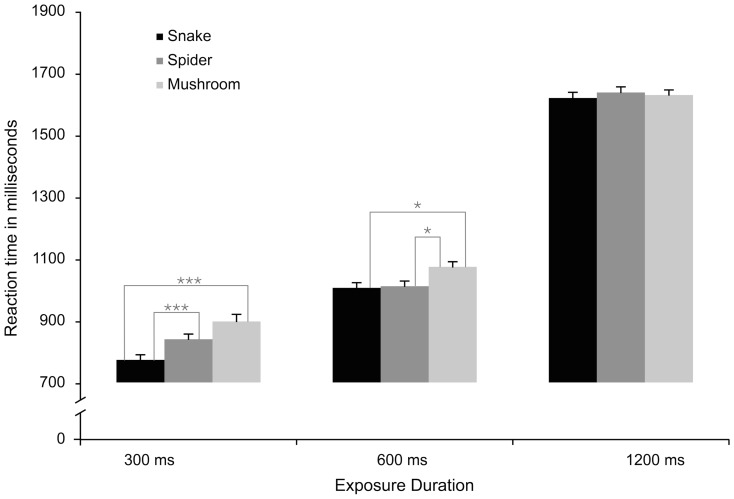
Experiment 1: Mean Reaction Times (RTs) in milliseconds (ms) to locate a discrepant target stimulus that could be a snake, a spider, or a mushroom, in displays exposed for 300 ms, 600 ms, and 1200 ms.

As shown by the significant interaction between target and exposure time, *F* (4, 204) = 19.54, *p*<.0001, the results confirmed our hypothesis that the detection of snakes was faster than that of spiders and mushrooms specifically at the shortest stimulus duration (see Fig. 2), independently on the number of distractor stimuli. When the stimulus duration was 300 ms, Tukey tests showed that snakes were detected faster than both spiders and mushrooms (both *p*_s_<.0001). With a duration of 600 ms, snakes and spiders did not differ but were both faster than mushrooms (both *p*s<.05), and when the duration was 1200 ms there were no reliable difference between the different target categories.

In addition to the reliable interaction between stimuli and exposure duration described in the article, there were several other reliable effects in the ANOVAs. Participants were overall faster to detect snakes (*M* = 1079 ms), compared to mushrooms (*M* = 1162 ms), *F* (2, 102) = 46.89, *p*<.0001, *η_p_^2^* = .48, with spiders in between (*M* = 1116 ms), marginally (*p* = .08) faster than mushrooms but not different from snakes. The analyses of the RT data also showed that, in general, participants were slower to detect the target stimulus in the large (8 items) set size (*M* = 1139 ms), compared to small one (4 items) (*M* = 1098 ms), *F* (1, 51) = 37.43, *p*<.0001, *η_p_^2^* = .41. Moreover, RTs were increased at longer exposure durations (*M* = 1628 ms), compared to medium (*M* = 1029 ms) and short durations (*M* = 835 ms), *F* (2, 102) = 1724.1, *p*<.0001, *η_p_^2^* = .97. These effects significantly interacted with each other, *F* (2, 102) = 4.74, *p*<.05, *η_p_^2^* = .08, which could be attributed to a significant difference between set sizes at 300 ms (*p*<.05), but not at 600 ms and 1200 ms.

#### Accuracy

**Figure 3:**
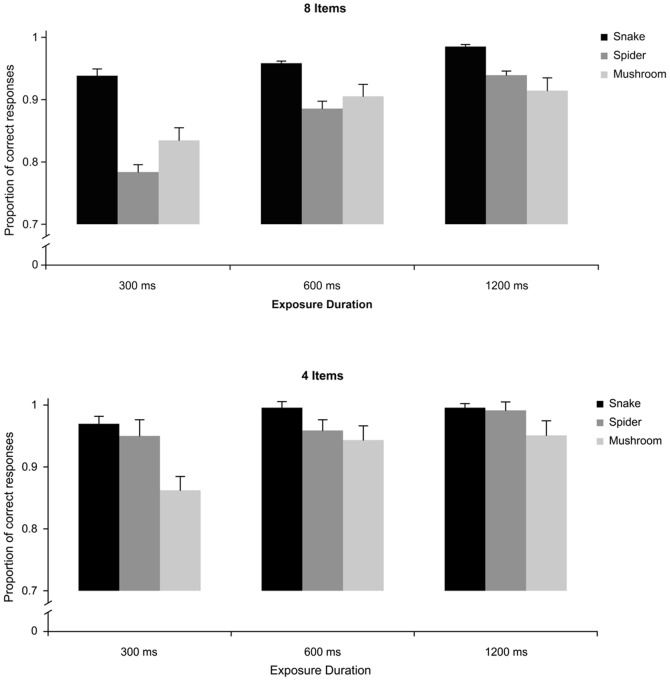
Experiment 1: Mean accuracy proportions to locate a discrepant target stimulus that could be a Snake, a Spider, or a Mushroom, in displays exposed for 300 ms, 600 ms, and 1200 ms that included eight (a) or four items (b).

The results from the accuracy data concurred with those from the RT data with one important addition. In contrast to the RT data, the three-way interaction between target, exposure duration, and set size was also significant, *F* (4, 204) = 6.69, *p*<.0001, suggesting that the difference between snakes and spiders was larger with many rather than few distractors (see Fig. 3). More specifically, the detection of snakes was more accurate than detection of spiders and mushrooms, particularly at the shortest stimulus duration (300 ms) and when embedded among 8 rather than 4 distractor pictures (fruits). Snakes were more accurately detected than spiders (and mushrooms) only with 8 item stimulus set (Fig. 3, upper panel), and the 300 and 600 ms duration stimuli (*p*<.001 for both comparisons). Snakes were always more accurately detected than mushrooms (*p*<.05), whereas spiders were more accurately detected than mushrooms only for the 300 ms-small display condition (Fig. 3, lower left panel).

The reliable three-way interaction between target, exposure duration and set size described in the article was related to several two-way interactions. The first one concerned type of target and exposure duration, *F* (4, 204) = 5.14, *p*<.001, *η_p_^2^* = .08, and was attributable to larger accuracy of snakes than spiders and mushrooms at the 300 ms exposure (with set size collapsed). Second, the interaction between target and set size, *F* (2, 102) = 15.60, *p*<.0001, *η_p_^2^* = .21 reflected larger difference between snakes and the other stimuli with the large than the small stimulus set (exposure duration collapsed). Finally, the significant interaction between exposure duration and set size, *F* (2, 102) = 7.58, *p*<.001, *η_p_^2^* = .10, suggested that the accuracy for exposure durations (target collapsed) tended to increase with increasing exposure duration with the large set size whereas it remained more stable with the small set size.

In addition, participants were overall more accurate to detect a snake target (*M* = 97%), compared to mushroom and spider targets (*M* = 90%, and *M* = 92%, respectively), *F* (2, 102) = 16.12, *p*<.0001, *η_p_^2^* = .24, although post hoc Tukey tests only revealed a marginal difference between snake and mushroom target stimuli (*p* = .07). The overall accuracy increased with an increase in exposure duration, with the percentage of correct responses being significantly higher in the 1200 ms condition (*M* = 96%) and 600 ms conditions (*M* = 94%), compared to displays presented for 300 ms (*M* = 89%), *F* (2, 102) = 58.71, *p*<.0001, *η_p_^2^* = .49. Moreover, accuracy was also lower in the large (*M* = 91%) than the small (*M* = 96%), displays *F* (1, 51) = 59.08, *p*<.0001, *η_p_^2^* = .46.

## Experiment 2: Superior detection of snakes in peripheral vision

This experiment tested whether the detection of snakes is especially superior to that of spiders and mushrooms in peripheral vision, given the advantage of an enlarged field of view for detecting nearby snakes and the hypothesis that snakes may stimulate mainly the magnocellular system. The spatial distribution of targets and distractors in this experiment changed randomly from trial to trial, and the location of the target was systematically varied between foveal (<1.2°), parafoveal (3.4°), and peripheral (5.7°) locations.

Our primary attention measure in this experiment was attentional efficiency, which is defined by the slope of the regression line for correct RTs to identify targets, as a function of the number of items (set size) in the display. This measure provides estimates of the increase in RT with each added item in the display; efficient search is indicated by a slope coefficient <10 ms/item [35].

### Apparatus

The apparatus was the same as for Experiment 1.

### Stimuli

**Figure 4:**
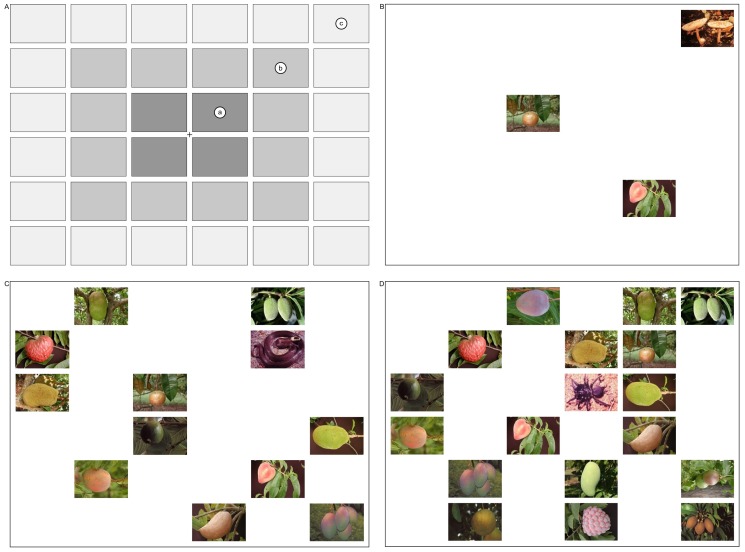
The visual display in Exp. 2 was presented in a grid, with the pictures arranged on an imaginary rectangle that was divided into a 6×6 grid (i.e., 36 cells). Upper left: Arrangement of the images in the display in the four foveal locations (A), twelve parafoveal locations (B), and twenty peripheral locations (C) (1.2°, 3.4°, and 5.7°, respectively) in Exp.2. Upper right: Example of a display with 3 items and a target picture (mushroom) in the periphery. Bottom left: Example of a display with 12 items and a target picture (snake) in the parafovea. Bottom right: Example of a display with 18 items and the target picture (spider) in the fovea.

The stimuli were the same as used in Experiment 1. The stimuli were randomly assigned to all the possible positions on the imaginary rectangles that divided the screen into a 6×6 grid (i.e., 36 cells) (see Fig. 4). The assignment to the different target types and set sizes was also performed randomly. Depending on set size, a different number of rectangles were filled with pictures. The size of each picture was 5.0×3.5 cm (150×100 pixels, with 71 Dots Per Inch [DPI]), distanced from each other by 0.5 cm, with the total size of the display being 32.5 cm (width)×23.5 cm (height).

### Task and Procedure

Target location was randomized and could occur at one of 4 (fovea), 12 (parafovea) or 20 (periphery) positions (Fig. 4). Blank spaces occupied the locations where no pictures were presented. Participants were exposed to 288 trials (displays with or without target), three types of target (snakes, spiders, and mushrooms), three possible spatial locations for the target (eccentricities of 1.2°, 3.4°, and 5.7°), four set sizes (3, 6, 12, 18), and four replications of both target present and target absent trials.

### Results and Discussion

#### Slopes

**Figure 5:**
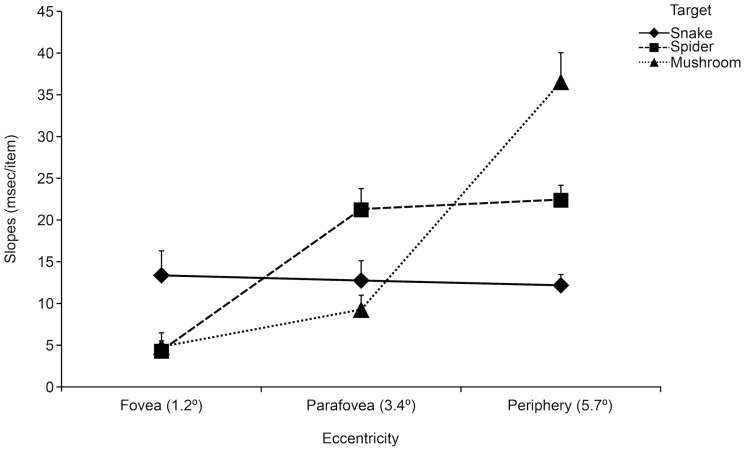
Experiment 2: Attentional efficiency reflected in slopes across different set sizes (3, 6, 12, 18) (expressed as the mean search time [in milliseconds]/searched item) for locating the target picture (snake, spider, mushroom) as a function of eccentricity (fovea, parafovea, periphery).

Consistent with our hypothesis, slope data showed a strongly significant interaction between targets and eccentricity, *F* (4, 164) = 25.06, *p*<.0001, *η_p_^2^* = .38. As predicted, the attentional efficiency for detecting snakes among distractors was unaffected by the eccentricity of target presentation. In contrast, attentional efficiency deteriorated with more peripheral presentation for spider and mushroom targets. As a result, at the most peripheral target location, snakes were more efficiently detected than spiders (*p*<.01) and spiders were more efficiently detected than mushrooms (p<.001). Even though it missed statistical significance for mushrooms, spiders showed more shallow slopes than snakes (p<.05) at foveally presented targets, which contributed to the interaction between target and eccentricity (Fig. 5).

There was also a main effect of targets on slopes *F (*2, 82) = 4.34, p<.05, *η_p_^2^* = .10 (p<. 05), with more shallow slopes for snakes (*M* = 13 ms/item) than for spiders (*M* = 16 ms/item) and mushrooms (*M* = 17 ms/item). Slope data also showed overall more efficient search in the foveal (*M* = 8 ms) than in the peripheral locations (*M* = 24 ms) (Tukey HSDs, *p*<.0001) and also more efficient search in the parafoveal (M = 14) than in the peripheral locations (Tukey HSD, *p*<.01), *F* (2, 82) = 45.75, *p*<0001, *η_p_^2^* = .53.

#### Reaction time

**Figure 6:**
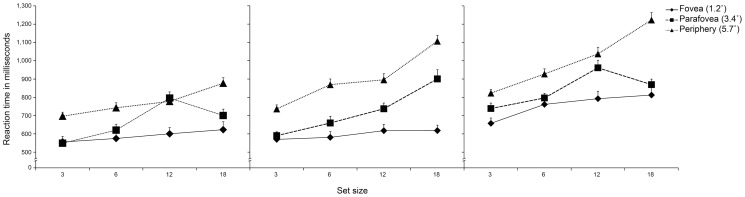
Experiment 2: Reaction times (RTs) in milliseconds (ms) to detect the target picture - snake (left panel), spider (middle panel), and mushroom detection (right panel), as a function of set size (3, 6, 12, 18) and location in the visual field (fovea, parafovea, periphery).

Direct analyses on RTs concurred with those from the slope measure in showing a reliable interaction between target, set size and eccentricity, *F* (12, 492) = 8.23, *p*<.0001, *η_p_^2^* = .17, which confirmed that participants detected snakes more quickly than spiders (and mushrooms) particularly in peripheral vision, compared to foveal locations, and with larger set sizes (*p*s<.0001) (see Fig. 6).

Moreover, the two-way interactions between target and set size, *F* (6, 246) = 5.93, *p*<.0001, *η_p_^2^* = .13, and between target and eccentricity, and *F* (4, 164) = 10.23, *p*<.0001, *η_p_^2^* = .20, indicated that participants detected snakes faster than they detected spiders and mushrooms, particularly in displays with more items (18 pictures) (Tukey HSDs, *p*<. 001), and in the peripheral as compared to foveal locations (Tukey HSDs, *p*<.001).The results showed that RTs for the detection of fear-relevant target stimuli (snakes and spiders) (*M* = 669 ms; *M* = 724 ms, respectively), were overall shorter than those to neutral stimuli (mushrooms) (*M* = 855 ms) (Tukey HSDs, *p*<.01), *F* (2, 82) = 131.03, *p*<.0001, *η_p_^2^* = .76, for the main effect of target. Independently of the fear-relevance of the stimuli, RTs were overall longer in response to targets presented in peripheral visual fields (*M* = 880 ms), as compared to the ones presented in the fovea (*M* = 642 ms) and parafovea regions (*M* = 733 ms) (Tukey HSDs, *P*<.01), *F* (2, 82) = 385.38, *p*<.0001, *η_p_^2^* = .90 for the main effect of eccentricity. The displays with a large number of distractors (12, 18) also resulted in a general increment in RTs (*M* = 790 ms; *M* = 838 ms, respectively), as compared to the displays containing fewer distractors (3, 6) (*M* = 651 ms; *M* = 716 ms, respectively) (Tukey HSDs, *p*<.05), *F* (3, 123) = 160.69, *p*<.0001, *η_p_^2^* = .80. However, this increase in RTs with the larger number of distractors was significantly steeper for the parafoveal and peripheral locations, *F* (6, 246) = 21.78, *p*<.0001, *η_p_^2^* = .35 (see Fig. 6).

For the target-absent RT trials we ran a repeated measure ANOVA, with the set size (3, 6, 12, 18) as the within-participants factor. The results showed that RTs were significantly shorter for displays with 3 distractors (*M* = 827 ms), followed by displays with 6 (*M* = 1070 ms), 12 (*M* = 1419 ms) and 18 distractors (*M* = 1706 ms) (Tukey HSDs, *p*<.0001), *F* (3, 123) = 335.36, *p*<.0001, *η_p_^2^* = .91, for the main effect of set size.

#### Accuracy

The overall performance in locating the target pictures decreased with eccentricity, with the percentage of correct responses significantly dropping in the peripheral (*M* = 90.63%) and parafoveal conditions (*M* = 91.25%), compared to foveal presentation of targets (*M* = 97.70%), *F* (2, 82) = 39.20, *p*<.0001, *η_p_^2^* = .49.

## Experiment 3: Detection of task-irrelevant snakes

Because the task of the previous experiments required active top-down controlled search for a target defined as discrepant from the background, they do not address the important issue of automatic snake detection when active attention is otherwise focused in the environment. Accordingly, Experiment 3 tested the hypothesis that snakes would automatically attract attention when they are irrelevant for the search task, and particularly when they are embedded in cluttered scenes (i.e., larger set size). Although a recent study showed that snakes (compared to spiders and mushrooms) produce more interference in an analogous visual search task [33], we incremented the complexity of the perceptual load of the task to test whether the snake interference advantage prevailed. Thus, along with the number of items in the display, we further manipulated one of the important factors known to increase load, i.e., redundancy or the similarity of the background stimuli [33], [36]–[39] In the homogeneous condition, the fruit pictures were identical, which facilitated their identification and left more resources for efficient detection of the critical extra distractors. In the heterogeneous condition, all fruit pictures were different and had to be individually discarded, presumably leaving less resource for processing of the critical distractors. Attention capture was assessed by comparing RT to target detection for displays with and without a task-irrelevant distractor.

### Apparatus

The apparatus was the same as for Experiments 1–2.

### Task and Procedure

Unlike Experiment 1–2, in Experiment 3 the participant's task was to determine, as quickly and accurately as possible, whether a specified target stimulus (a bird) was present or absent among the background distractor stimuli. They were also informed that at some trials a snake, a spider or a mushroom would replace one of the background pictures, although the task was always to decide upon the presence or absence of the designated target. Half of the displays included 4 or 6 background stimuli (pictures of fruits; target absence trials), and the other half included a target (a picture of a bird) against a background of 3 or 5 pictures of fruits (target present trials).

Participants were presented with 192 trials (96 target present and 96 target absent trials), randomly assigned to each participant. On 36 of both target present and target absent trials a distractor picture replaced one of the background stimuli for both display sizes, with the equal probability of appearance for each of the distractor categories. On the remaining 60 trials no distractor stimulus was presented. Both targets and distractors positions were counterbalanced across trials. Within displays, the background stimuli could be homogeneous or heterogeneous, which was manipulated as a between-participants factor. In addition, the participants could be exposed to two different stimulus set sizes (4; 6), manipulated as a within-participants factor. The additional within-participants factor was the type of distractor stimuli (snake, spider, or mushroom), with displays with no distractor picture as the control condition.

### Results and Discussion

#### Reaction time

**Figure 7:**
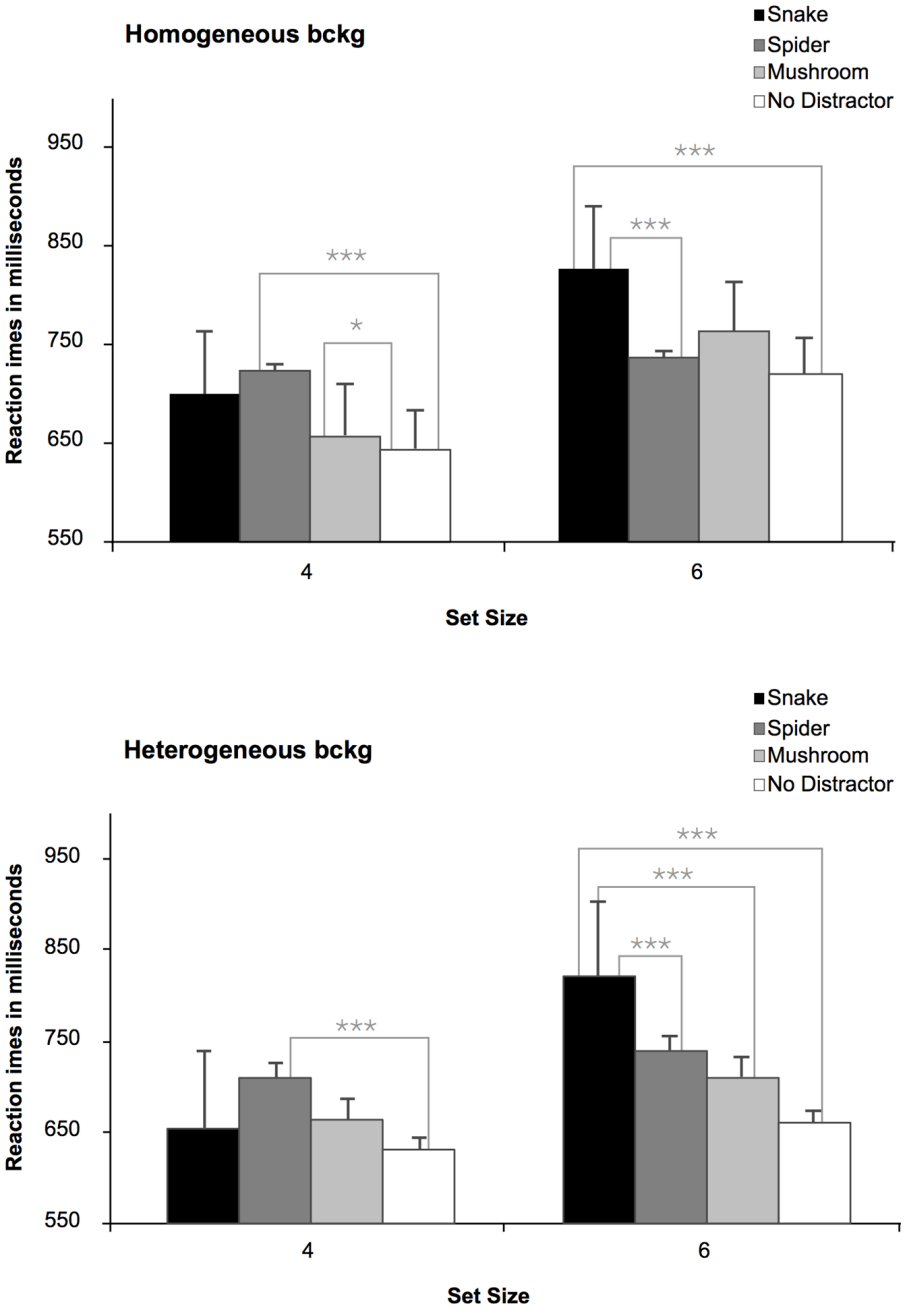
Mean Reaction Times (RTs) in milliseconds (ms) to locate a discrepant target (bird) in the different type of distractor conditions (snake, spider, mushroom, and no distractor), in Experiment 2. The upper panel refers to the homogeneous displays, whereas the lower panel illustrates the heterogeneous displays, both as a function of the set size (4; 6). Longer RTs indicate larger interference scores.

The hypothesis of more interference by snakes than by spiders and mushrooms with increasing perceptual load is most directly tested by the interaction between type of distractor, distractor homogeneity, and set size, *F* (3, 165) = 3.77, *p*<.05. When the stimulus set contained 6 items, snakes were the only distractor stimulus that produced a significant interference effect, as shown by a reliable slowing of RTs compared to the basic condition with only the background stimuli (Fig. 7, right parts). In addition, with the large stimulus set, snakes produced reliably more distraction than spiders both with homogenous and heterogeneous stimulus sets, whereas spiders and mushrooms did not produce any reliable evidence of interference effects. This is in marked contrast to the results with the small stimulus sets, in which spiders appeared to produce more interference than snakes (no difference for homogeneous background stimuli, and barely reaching significance, *p* = 0.05, for the heterogeneous background) (Fig. 7, left parts).

These findings were further clarified by the highly significant interaction between type of distractor and set size, *F* (3, 165) = 16.11, *p*<.0001. Collapsing across homogenous/heterogeneous background stimuli in the 6-distractors display, snakes produced clearly more interference than spiders, mushrooms, and no distractor displays (all *p*s<.0001). In contrast, with the 4-item displays, again collapsing over backgrounds, spider distractors produced enhanced interference compared to snake (*p*<.05), and mushroom distractors (*p*<.001), and displays with no distractor stimulus (*p*<.0001).

Finally, the results showed that, in general, there was a larger interference (longer RTs) for displays including a fear-relevant stimulus (snake and spider distractors), than a neutral one (mushroom) (*p*<.001), *F* (3, 165) = 36.03, *p*<.0001. However, the difference in interference between spider and mushroom distractors missed statistical significance (*p* = .06). Finally, there was an overall larger interference from distractors when the displays were larger (*M* = 746 ms), compared to when they were smaller (*M* = 673 ms), as illustrated by the main effect of set size, *F* (1, 55) = 86.47, *p*<.0001.

Thus, consistent with an evolutionary perspective, attention appeared to be automatically reoriented to suddenly appearing snakes in the immediate environment, which goes in line with the preliminary findings from [33].

#### Accuracy

The analysis of detection accuracy showed that displays with six items snake (*M* = 93%) and spider distractors (*M* = 94%) produced more interference than displays with no distractor picture (*M* = 99%) (*p*<.05). For displays with four items, however, there were no evidence of significant differences from the fear-relevant distractors, which resulted in a reliable interaction between the type of distractor and set size *F* (3, 165) = 6.92, *p*<.001, *η_p_^2^* = .11. The results also showed that larger displays (set size of 6) were associated with lower accuracy (*M* = 96%), compared to displays with fewer items (*M* = 98%), *F* (1, 55) = 7.97, *p*<.01, *η_p_^2^* = .13. The homogeneity of the background stimuli did not produce effects on the accuracy data.

## Experiment 4: Detection of snakes is independent of perceptual load

The consistent finding that more effective detection of snakes than other stimuli in Experiments 1–3 was restricted to perceptually demanding contexts, suggest that snake detection is insensitive to, or even independent of, perceptual processing resources (e.g., [34], [40]. Attention to spiders and neutral stimuli, on the other hand, which deteriorated with increased demand for perceptual resources, as manipulated with set size, conform to established attention theory [34] and a large body of data demonstrating a strong negative influence of number of background distractors on visual search performance [35], [37]. Thus, according to our data so far, snakes – but not spiders – may be automatically detected. The purpose of Experiment 4 was to directly confirm this hypothesis with a paradigm developed for assessing automatic detection when perceptual resources are fully engaged by a highly demanding primary task [41].

Because the subject population in the previous three experiments was dominated by females, the majority of participants in these experiments also were females. Thus, generalization of the conclusions to males must be hedged by caution. To address possible sex differences in snake detection, we therefore incorporated close to an equal number of females and males in the present sample (see Methods: Participants).

### Apparatus

A 19″ CRT monitor, with a screen resolution of 1024×768 pixels and a refresh rate of 85 hz connected to a PC was used for stimulus presentation. Viewing distance was approximately 60 cm. Participants used adjacent buttons (“1” and “2”) on the numerical keyboard for their responses.

### Stimuli

**Figure 8:**
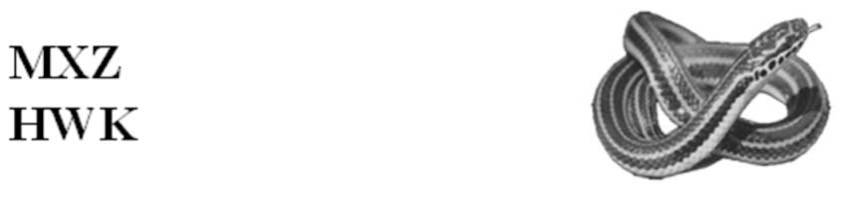
Example display of Experiment 4, depicting a high perceptual load trial with a snake distractor. Note that stimuli are not drawn to scale.

We used a different set of grayscale images depicting snakes, spiders, flowers and mushrooms that were equalized so that mean number of pixels, mean luminance and mean contrast (i.e., standard deviation of luminance) did not differ among the four categories (see S1 File). They were presented against a white background in randomized order. Experiment 4 used a central task with very brief (47 ms) stimulus duration, in which participants had to rapidly decide the identity of a target letter at the center of the display. In the low perceptual load condition the target was presented alone, and in the high load condition it was presented among several other letters (see [40]). On 20% of the trials, a task-irrelevant distractor picture (snake, spider, flower or mushroom) was presented simultaneously with, but completely outside of the spatial area covered by, the central task (see Fig. 8). The distractor pictures were in gray-scale and controlled for brightness and contrast. More details about the stimuli are given in S1 File.

### Task and Procedure

Participants performed a two-choice reaction time task, in which they were to indicate, as quickly and accurately as possible, the identity of a target letter (X or N). The target letter was presented alone (low perceptual load) on 50% of the total number of trials or surrounded by 5 non-target letters (high perceptual load) arranged in two 3-letter strings directly above and below fixation on the remaining trials. The position of the target letter was randomized over the six possible positions surrounding fixation on every trial. Each trial started with the presentation of a fixation cross against white background (750 ms), followed immediately by the stimulus display (47 ms = 4 refresh cycles at 85 Hz), and a blank white response screen (700 ms). On 20% of the trials, a task-irrelevant distractor image depicting a snake, spider, flower or mushroom was displayed peripherally, either horizontally or vertically from fixation (image center 9.5° from fixation), simultaneously with the stimulus display (Fig. 8). The order of both conditions (perceptual load level, image distractor presence) and stimuli (image distractor type, target letter, non-target letters) was fully randomized for each participant. Participants initially completed 70 practice trials (without image distractors) with accuracy feedback, followed by 3 blocks of experimental trials (240 trials/block). A participant-paced pause interval occurred between each block.

### Results and Discussion

#### Reaction Time

**Figure 9:**
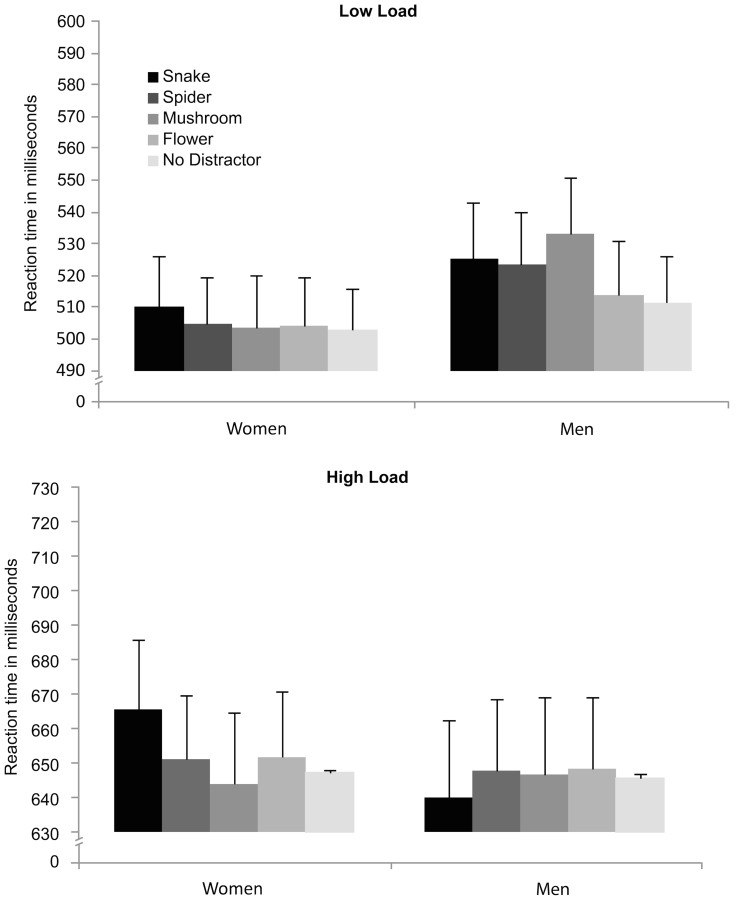
Experiment 4: Mean Reaction Times (RTs) in milliseconds (ms) to discriminate the target letter (X or N) in the different type of distractor conditions (snake, spider, mushroom, flower, and no distractor), as a function of the perceptual load condition (low; high) and the sex of the participant (women; men). Longer RTs indicate larger interference scores.

A mixed effects repeated measures ANOVA was conducted for choice RT, with perceptual load (low/high) and distractor (no/flower/mushroom/snake/spider) as within-participant factors, and gender as between participants factor. The results showed a very robust main effect of perceptual load, with slower overall RTs at high load (*M* = 649 ms) than low load (*M* = 513 ms), *F*(1,47) = 439.26, *p*<.0001, *η_p_^2^* = .90, confirming the effectiveness of the perceptual load manipulation. There was no main effect of gender either for accuracy or RT (p>.17), indicating that the overall performance of males and females did not differ (see results for accuracy analysis). Furthermore, there were no main effect of distractor (*p*>.05), and no interaction effect of perceptual load and distractor (*F*<1). The critical simple contrasts comparing each distractor type against the no-distractor baseline however revealed an effect of snakes, *F*(1,48) = 7.51, *p* = .009, *η_p_^2^* = .14, confirming that snakes reliably distracted from performance on the central task (Snake distractor: *M* = 585 ms; No distractor: *M* = 577 ms), regardless of available perceptual processing resources. Furthermore, contrasts revealed a three-way interaction between gender, snake distraction and high perceptual load, *F*(1,47) = 5.68, *p* = .021, *η_p_^2^* = .11, indicating that the effect of snake distraction on RT was particularly pronounced for women and at high perceptual load (*p*<.05) (Fig. 9). No other distractor type interfered with the target task during high perceptual load.

To our knowledge, this is the first unequivocal report of threat-related attention capture during high perceptual load, despite a wealth of research on the subject (e.g., [42]–[45]). Importantly, the load was sufficient to block distractive effects of spiders but not of snakes.

#### Accuracy

Response accuracy was similarly analyzed, where proportion correct was submitted to a 2 (Perceptual load: high/low)×5(Distractor: no/flower/mushroom/snake/spider)×2(Gender) mixed effects repeated measures ANOVA. The analysis showed a main effect of perceptual load analogous to the effect on RT, *F*(1,47) = 242.661, *p*<.0001, *η_p_^2^* = .838, with lower accuracy during high load (*M* = 68%) than during low load (*M* = 92%), no main effect of distractor (*p* = .15), no perceptual load by distractor type interaction (*F*<1), and no gender effects (*p*s>.17).

## General Discussion

Our results consistently show that snakes were more effectively detected than spiders and neutral stimuli, particularly in situations with high demands on perceptual processing. Inspired by Isbell's SDT [8] we formed hypotheses about crucial ecological conditions likely to modulate snake detection on the premise that snakes have provided deadly dangers throughout primate evolution. Because of their frequent use of ambush hunting strategies [14], [16] we assumed that effective defense would be predicated on efficient detection in order to break snake camouflage. Our results show a predicted series of reliable statistical interactions to the effect that snakes surpassed spiders and mushrooms in stimulus conditions where lurking snakes would be hard to detect. They included brief exposures (Experiment 1), peripheral stimulus presentations (Experiment 2), and cluttered displays (Experiments 1–3). Perhaps most interestingly, only snakes captured attention automatically when presented as task-irrelevant distractors for participants engaged in cognitively demanding tasks (Experiments 3–4). Snake detection, therefore, appears resistant to well-known factors that impede detection performance in visual search settings, such as many background distractors [32], [33], [35], [37], peripherally presented targets [46] and elevated perceptual load [32], [33], [40], [42].

In contrast, spiders were more efficiently detected than snakes when presented foveally (Experiment 2). In this condition the task simply required detection and identification of the target without any need either to disengage attention from the fixation cross or to move it to the spatial location of the target [47]. The efficient detection of foveally, but not peripherally, presented spider targets suggest that some highly diagnostic piece of information was available at foveal, but not at peripheral vision. Visual acuity is at its optimum in the fovea but sharply and nonlinearly decline towards the visual periphery [48]. Foveal ganglion cells primarily project to the cortex via the dense, slow conducting parvocellular pathway, while the retinal periphery projects via the low resolution, but rapidly conducting magnocellular pathway [49]. For example, the legs of spiders might be less salient in low resolution peripheral vision, because they require the high visual acuity mediated by the parvocellular pathways for analysis (e.g., [50]). In contrast, the salience of snake features appear to be relatively invariant across the visual field, as indicated by the flat RT slopes in Experiment 2 (see Fig. 5), presumably reflecting magnocellular projections from the retinal periphery. Importantly, these differences pertain to the natural objects (i.e., snakes and spiders), and not to idiosyncrasies of the stimulus material. The images depicting snakes and spiders, respectively, did not differ in spatial frequency power at any range of the frequency spectrum (see SEM). In addition, in the final experiment (Experiment 4), stimuli were drawn from a different set of images controlled for luminance and contrast and presented devoid of background distractors.

Salience invariance across the visual field would facilitate the reflexive reorienting of spatial attention to unattended snakes. The critical neural locus for reflexive shifts of both covert and overt (i.e., eye movements) attention based on retinal innervation (i.e., bottom up salience) [49] is the superior colliculus, which predominantly receives input from the magnocellular stream [51]. The superior colliculus is also heavily implicated in automatic or unconscious attention to threatening information (e.g., fearful facial expressions), which it transmit to the amygdala [52]. Highly salient stimuli can trigger superior colliculus-initiated saccades within 120 ms of stimulus onset [49], [53]. The pulvinar, a higher level center of neuronal control, directly connects with the superior colliculus for attentional guidance [54]–[56]. Moreover, the pulvinar also regulates the information transfer during these automatic, preattentive processes [52], [57]. Thus, salient features of unattended snakes should thereby be sufficient to rapidly both [i] elicit reorienting of attention via the superior colliculus and the pulvinar, and [ii] activate defensive responses (e.g., freezing) via the amygdala central nucleus (e.g., [52]).

The high temporal resolution provided by magnocellular vision offer an account also for the preferential detection of snakes at short (300–900 ms), but not long (1200 ms) exposures of the search display (Experiment 1) [58], which is consistent with the results of a previous study from our lab [33]. Although in Soares and Esteves (2013) study we only compared very short exposure durations (150 ms, 300 ms), which could have explained the lack of differences in target detection as a function of exposure durations, we did obtain results that were consistent with the hypothesis that snakes are detected under more complex conditions. More specifically, snakes targets were overall detected more accurately than spider targets, and this snake advantage effect was more clear-cut with many (high load) than with few distractors (low load), independently of the exposure duration (150 or 300 ms). Finally, the attentional capture by snakes presented for a mere 47 ms in the visual periphery (Experiment 4) can also be accounted for by magnocellular processing. Task-irrelevant distractors that activate the magnocellular system capture attention more strongly than parvocellular-activating distractors, and the magnocellular-based attentional capture is less susceptible to top-down control [53], which provides a mechanism for why task-irrelevant snakes, but not spiders, captured attention in the present experiments (Experiments 3–4). Thus, our data are consistent with the proposition that snake detection is mediated by the magnocellular visual pathways, and possibly involve sub-cortical structures, such as the superior colliculus and amygdala, known to be important for rapid processing of threat-related cues across species [17], [20], [52]. Detection of mammalian predators by primates appear to be driven by simple cues, such as dark spots against a brighter background in the case of leopards [59], but similar data for snake detection are presently scarce (but see e.g., [60]). Hence, it remains an important challenge to identify these critical features.

The dissociation between detection of snakes and spiders observed in our experiments is consistent with the SDT [8] argument that the superior, if not automatic, detection of snakes in a visually demanded context is a specialized anti-predator adaptation. The conclusion that snake detection is an evolutionary adaptation is further supported by reports of superior detection of snakes in visual search settings in small children [61]–[63] and in lab-reared, snake-naïve rhesus monkeys [64] which suggests that the attentional priority of snakes does not depend on prior experience. However, the results from these studies must be regarded as tentative because they did not include any comparison with spiders, which was a critical feature in our series of experiments.

It is interesting that the differences between snakes, on the one hand, and spiders and neutral stimuli, on the other, were reliably clearer for female than for male participants in Experiment 4. This is consistent with the higher reported prevalence of snake phobias in females than in males [65]. Because Experiments 1–3 mostly included women, strong conclusions based on gender differences should await a larger scale replication study. Moreover, and given our goal of having a continuous variation in the fear levels, the sample selection in Experiments 1–3 may have resulted in an overrepresentation of fearful participants relative to the general population, which may possibly pose a limitation to the generalizability of our results. Yet, in Experiment 4 the sample was randomly selected and the general pattern of results was highly consistent with the previous experiments. Additionally, and because we used university students throughout the four experiments, future studies with more representative samples are warranted.

By pointing to a quite unique relationship between human attention mechanisms and the detection of snakes, our data are consistent with some of the basic behavioral implications of the SDT. The pattern of findings across experiments, furthermore, suggests that the detection of snakes but not that of spiders reflect automatic detection routines of likely evolutionary origin. By supporting the SDT, our data provide new perspectives on the potentially unique role of snakes as agents likely to have shaped central aspects of primate evolution, including what has been regarded a hallmark of African apes, our superb vision. However, so far this is merely a modest step in the extensive undertaking of empirically evaluating this rich theory, which integrates findings across a wide array of scientific disciplines.

## Supporting Information

S1 File **The Hidden Snake in the Grass: Superior Detection of Snakes in Challenging Attentional Conditions.**
(DOCX)
