# Supplementary material for: The Hidden Snake in the Grass: Superior Detection of Snakes in Challenging Attentional Conditions
Source: PLoS One. 2014 Dec 10;9(12):e114724. doi: 10.1371/journal.pone.0114724 (PMC4262429; doi:10.1371/journal.pone.0114724)
Supplement: S1 File — The Hidden Snake in the Grass: Superior Detection of Snakes in Challenging Attentional Conditions. (DOCX) [file pone.0114724.s001.docx]

**Supporting Information S1**

**The Hidden Snake in the Grass: Superior Detection of Snakes in Challenging Attentional Conditions**

Sandra C. Soares^1, 2, 3^, Björn Lindström^4^, Francisco Esteves^5^, Arne Öhman^4^

^1^ Department of Education, University of Aveiro, Portugal

^2^ IBILI - Institute for Biomedical Imaging and Life Sciences, Faculty of Medicine, University of Coimbra, Portugal

^3^ Center for Health Technology and Services Research (CINTESIS), Faculty of Medicine, University of Porto, Portugal.

^4^ Division of Psychology, Department of Clinical Neuroscience, Karolinska Institute, Stockholm, Sweden

^5^ Department of Psychology, Mid Sweden University, Sweden

**Corresponding author:**

Sandra C. Soares

University of Aveiro

Department of Education

Scientific Area of Psychology

Campus Universitário de Santiago

3810-193 Aveiro

Portugal

Email: [sandra.soares@ua.pt](mailto:sandra.soares@ua.pt)

**Data Reduction**

The analysis of RT excluded error trials. In addition, outliers greater than ± 3 * standard deviations (SD) were replaced by the individual’s mean ± 3 * SD. In order to meet the requirements of normally distributed data, reaction times were logarithmically (log10) transformed prior to statistical analyses, although values given in the text and in the figures were back-transformed to RTs (in ms) to facilitate interpretation of the data. The slope coefficient [1] was calculated based on a least-square estimated regression line of RT as a function of set size for each participant and experimental condition. Follow-up tests were accomplished using Tukey HSDs or planned contrasts. Significance levels were set at *p* < 0.05, and partial *η^2^* (*η_p_^2^*) is used as estimate of effect sizes.

**Analysis of stimuli material**

**Luminance and RMS contrast.**

To investigate if low level image features of the stimuli used in Experiments 1-3 (the stimuli set used for Experiment 4 was equalized on luminance and contrast) might have contributed to the results, additional analyses were conducted. Snake, spider and mushroom color stimuli were converted to grayscale using the standard Matlab algorithm (rgb2gray; Matlab, 2010a, The Mathworks, Natick, MA.), where after mean stimulus intensity (luminance) and root mean square (RMS) contrast [2] was calculated for each image. Separate one-way ANOVAs were conducted for each measure with Tukeys HSD as follow-up test. The ANOVA of luminance [*F*(2,51) = 5.94, *p* < .01] showed that snakes and spiders had higher mean luminance than mushrooms (*p*s < .01), while there was no luminance difference between snakes and spiders (*p* = .99). The ANOVA of RMS contrast [*F*(2,51) = 4.01, *p* < .05] showed that mushrooms (*p* < .01) and spiders (borderline; *p* = .06) had higher contrast than snakes , while spider and mushroom did not differ (*p* = .96) in contrast. In summary, the experimental results reported in the current article cannot be attributed to systematic differences in low level features of the stimuli, as contrast were lower for snake than for control stimuli, while snake and spider images did not differ in mean luminance (while higher than for mushroom images).

**Spatial Frequency.**

Comparison of spatial frequency energy across stimuli types was conducted with discrete wavelet transformation of the grayscale images [3]. The frequency spectrum was divided into four orthogonal bands (we used a low number of bands due to the small size [75x75 pixels] of the images used in Experiments 1-3) with the Haar wavelet function [3], where after the mean energy of each band was compared across stimuli types for each frequency band. Separate one-way ANOVAs were conducted for each frequency band.

**Experiments 1-3**. The ANOVA at the lowest frequency band [*F*(2, 52) = 0.9, *p* = .68] revealed no differences in spatial energy among stimuli types. Similarly, at the next frequency band, no differences were found [*F*(2, 52) = 2.25, *p* = .12]. At the next to highest frequency band, no differences were found [*F*(2, 52) = 0.37, *p* = .69]. At the highest frequency band, yet again no differences were found [*F*(2, 52) = 0.68, *p* = .51].

**Experiment 4.** At the lowest frequency band, the ANOVA [*F*(3, 1 55) = 57.3, *p* < .001], showed that spider images had higher energy than snake (*p* < .01), mushroom (*p* < .001), and flower images (*p* < .001), and snake images had higher energy than flower (*p* < .001) and mushroom (*p* < .001) while flower and mushroom images were comparable (*p* = .6). Similarly, at the next frequency band [*F*(3, 155) = 92.75, *p* < .0001], spider images had higher energy than snake (*p* < .01), mushroom (*p* < .001), and flower images (*p* < .001). Snake images had higher energy than flower (*p* < .001) and mushroom images (*p* < .001) while flower and mushroom images did not differ (*p* = .99). At the second highest frequency band [*F*(3,155) = 85.9, *p* < .0001], spider and snake images had higher energy than flower (*p*s < .0001) and mushroom (*p*s < .0001) images, while not differing from each other (*p* =.32). Flower and mushroom images did not differ in energy (*p* = .85). At the highest frequency band, spider and snake images likewise had higher energy than flower (*p*s < .0001) and mushroom (*p*s < .0001) images, while not differing from each other (*p* =.56). Again, flower and mushroom images did not differ in energy (*p* = .47).

In summary, in the stimuli set used for Experiments 1-3, there were no reliable spatial energy differences at any frequency band. Regarding the stimuli set used in Experiment 4, spider images had higher energy than both snakes and neutral images (mushrooms and flowers) in the lower two frequency bands, and snake images had higher energy than flower and mushroom images in the lower two frequency bands. At the higher frequency bands, snakes and spiders were comparable, both having higher spatial energy than mushroom and flower images. Critically, because snake stimuli had lower energy than spiders at the lower frequency bands, and were comparable at the two higher frequency bands, differences in spatial frequency can not account for the snake specific attention capture at high perceptual load reported in Experiment 4.

**Supplementary Information References**

1. Wolfe JM (1998) Visual search. In: Pashler H, editor. Attention. Hove, UK: Psychology Press. pp. 13-73.

2. Delplanque S, N´diaye K, Scherer K, & Grandjean D (2007) Spatial frequencies or emotional effects? A systematic measure of spatial frequencies of IAPS pictures by a discrete wavelet analysis. J Neurosci Meth ***165*: 144-150.**

3. Peli E (1990) Contrast in complex images. *J Opt Soc Am B: Opt Phys 7*: 2032-2040.
